# Magnetic properties of the complex concentrated alloy system CoFeNi_0.5_Cr_0.5_Al_x_

**DOI:** 10.1038/s41598-020-71463-3

**Published:** 2020-09-02

**Authors:** N. A. Morley, B. Lim, J. Xi, A. Quintana-Nedelcos, Z. Leong

**Affiliations:** Department of Materials Science and Engineering, University of Sheffield, Sir Robert Hadfield Building, Mapping St., Sheffield, S1 3JD UK

**Keywords:** Materials science, Structural materials, Applied physics

## Abstract

We study the change in magnetisation with paramagnetic Al addition in the CoFeNi_0.5_Cr_0.5_–Al_x_ (x: 0, 0.5, 1, and 1.5) complex concentrated alloy. The compositions were developed utilising the Mulliken electronegativity and d-electron/atom ratio. Spherical FeCr rich nanoprecipitates are observed for X: 1.0 and 1.5 in an AlCoNi-rich matrix. A ~ 5 × increase in magnetisation (from 22 to 96 Am^2^/kg) coincides with this nanoprecipitate formation—the main magnetic contribution is determined to be from FeCr nanoprecipitates. The magnetisation increase is strange as paramagnetic Al addition dilutes the ferromagnetic Fe/Co/Ni additions. In this paper we discuss the magnetic and structural characterisation of the CoFeNi_0.5_Cr_0.5_–Al_x_ composition and attempt to relate it to the interfacial energy.

## Introduction

The innovation of new materials has been deemed to be the key topic for development of new technologies in our modern world. For alloys, the traditional approach has been to consider the effect of multiple dilute additions to a larger alloy matrix. New complex concentrated alloys (CCAs), which are near-equimolar multiple element alloys (> 3) challenge this view and have pushed alloy research towards the centre of phase diagrams^1,2^.

Multiple alloying components mean that they can possess large property permutations owing to the vast compositional space that they cover. Considerable work has been done on understanding how compositional-structure affects mechanical properties. However, their functional properties are rarely the primary focus. As the base elements of CCAs are often CoFeNi (which is a known soft magnetic alloy), this can provide a basis for alloy design^1–5^. Our motivation in this work is to therefore investigate the potential of CCAs as soft magnetic materials.

Studies of CoFeMnNi + X, (X = Al, Cr, Ga, or Sn)^6^, CoFeCrNi-X, (X = Ga, Sn, Mn, or Al)^7^ show that Ga and Al additions tend to lead to the largest change in magnetisation in the system. Kormann et al*.*^8^ for CoFeNi-X alloys (X = Cr_A_Ag_B_, Cr_A_Au_B_, Cr_A_Pd_B_, and Cr_A_Cu_B_) also reported the deleterious effect of Cr on saturation magnetisation^9,10^. Also, the literature showed process-dependant saturation magnetisation dependence of CoFeCrNi–Al_x_ on post-processing that may be related to this, but in general for CoFeCrNi–Al_x,_ the phases change as x < 0.3 (FCC present), 0.5 < x < 0.7 (FCC + BCC present), and x > 0.9 (BCC present)^6,11,12^. FeCr-rich particles tend to form in a CoNiAl-rich matrix above x > 0.9, but the form of the particles depends upon the processing conditions^12^.

The influence of processing is important: in the CoFeCrNi system the magnetisation and Curie Temperature depends upon alloy fabrication processes (mechanical alloying vs. as-cast) due to increased Cr ordering (increasing its antiferromagnetic contribution)^13^. It has been shown that antiferromagnetic Cr clusters can display ferromagnetic behaviour when atomic-scale disorder is present^14^. The BCC and B2 structure is coherently present in CoFeCrNi–Al_x_^15,16^ and results in a change in strain and interfacial energy^17,18^. This may change the magnetisation of the composition as magnetic fields have been shown to affect interfacial energy in steel^19^, which have similar alloying elements to CoFeCrNi–Al_x_. Taken together, this suggests that to maximise saturation magnetisation in this composition, heat treatment to promote nucleation and growth of the disordered structure is required. This also homogenises the non-equilibrium phases from the arc-melting synthesis, providing a reference for future studies^1^.

Materials design strategies requires complex data analysis that either follow data driven discovery^20^ or functionally driven discovery^21,22^. In the latter desired target properties are used as an input to predict the require components that give rise to these properties. This shifts the focus to search and optimisation algorithms/strategies over the use of more general materials databases*.* In this work, we will be using the above identified targeted related properties to optimise the CoFeCrNiAl composition for higher saturation magnetisation at 300 K.

## Experimental section

### Synthesis

The samples were arc-melted at least thrice in the presence of a Ti getter in an Edmund Buhler Compact Arc Melter using components of > 99% purity. The samples were then cast into 6 mm diameter rods in a water-cooled copper hearth and were heat treated at 1,423 K for 10 h, and quenched in water. The as-cast samples were sectioned using a Struers Secotom-50 and mounted in bakelite. Mounted samples were ground and polished to a mirror finish.

### HF etching

The sample was etched with 3% HF in intervals of 5 s. Between each interval, the sample was observed under a microscope to determine the degree of etching. After etching, the samples were washed in isopropyl alcohol first, followed by washing with water.

### Characterisation

Etched surface of all samples were examined with an Olympus BX51 visible light optical microscope and overview images were obtained using mosaic function in Clemex software. Grain boundaries were not clearly visible despite the etching process and the grain boundaries were enhanced by tracing over visible grain boundaries at enhanced zooms so that microstructural features are more clearly observed. A Thermo Fisher Scientific Inspect F50 (20 kV) was used for SEM imaging. EDS line scans were carried out at sites with distinct segregation. X-ray diffraction (XRD) using a Bruker D2 phaser, with Cu K_α_ source was for XRD characterisation. From the XRD data, the lattice constants were determined using Bragg’s Law. The XRD data was converted with Powdll^23^.

Magnetic measurements: A MPMS-3 system was used to determine the magnetisation hysteresis loops and the field cooled (FC) magnetisation as a function of temperature and applied magnetic field. From the magnetisation hysteresis loops, the coercive fields and saturation magnetisations were determined. For the experimental step size, field measurements were taken on a log-step scale, to allow for more points around the low field region, compared to the high field region, where saturation occurs. Thus between – 3 kA/m and 3 kA/m the field step size was 0.5 kA/m or less. The range was 0.04 kA/m up to 0.5 kA/m at 3 kA/m. From the FC data, the Curie Temperatures were determined from the peak in the δM/δT vs. T plots.

Image analysis: Micrograph images shown are as taken, with the optical micrographs for n-Al_1.0_ and n-Al_1.5_ having the grain boundaries enhanced. This was performed by zooming in 10 × onto the taken micrographs and tracing along any observed grain boundaries by hand. The contrast between the grain boundaries and the grains were very low and due to the colour fluctuations in the optical micrographs, image histogram filters were unable to distinguish the grain boundaries for an algorithmic solution. No other modifications were made to the micrographs. Particle size was determined from image analysis and are made utilising the Mathematica package^24^ and its built-in image analysis tools. In order to determine the precipitate size, the micrographs to be analysed are first denoised using a suitable algorithm to preserve the edges. After that, the image was auto-adjusted according to its histogram data and binarised. Edge-detection is used to determine the perimeter of the features and from this its morphological components were determined. Histogram plots of the size distribution is done in order to determine the median and mean particle size distributions.

## Alloy design

Functionality driven discovery of CCAs use either *ab-initio* or semi-empirical approaches. Quick alloy design turnaround demands the development of faster semi-empirical approaches that can later be confirmed by *ab-initio*. Attempts to discriminate between compositions include using Hume-Rothery^25^ rules of valence electron concentration^26^, and the Miedema model^27^ to investigate solid-solution stability. Other predictive semi-empirical parameter schemas attempting to obtain more accurate results also exist^3–5,26,28–30^. A method correlating bond-length distortion to structural stability is used in this work^31^, with further details in the Supplementary Appendix A. The CoFeNi_0.5_Cr_0.5_Al_x_ composition satisfied our criteria, and so was selected. The results are compared, with good agreement, to enthalpy of mixing/valence electron predictions and are shown in Table1.

**Table 1:** Information on designed CoFeNi_0.5_Cr_0.5_–Al_x_ alloys, predicted phase stabilities, phases detected via XRD, lattice parameters from XRD, and micrograph grain sizes.

Sample	Name	Stoichiometry	Phase (Pred.)	Phase (Exp.)	Lattice constant	Grain size (μm)
CoFeNi_0.5_Cr_0.5_Al_0.0_	n-Al_0_	Co_33_Fe_33_Ni_17_Cr_17_	FCC	FCC	3.57	692
CoFeNi_0.5_Cr_0.5_Al_0.5_	n-Al_0.5_	Co_29_Fe_29_Ni_14_Cr_14_Al_14_	BCC/FCC	BCC/FCC	2.86/3.58	206
CoFeNi_0.5_Cr_0.5_Al_1.0_	n-Al_1.0_	Co_25_Fe_25_Ni_13_Cr_13_Al_25_	BCC/Com	BCC/B2	2.89	196
CoFeNi_0.5_Cr_0.5_Al_1.5_	n-Al_1.5_	Co_22_Fe_22_Ni_11_Cr_11_Al_33_	BCC/Com	BCC/B2	2.88	316

The heat treatment temperatures were determined by taking the difference between the free energy of mixing of the disordered phase and an ordered B2 phase consisting of Al/Co/Ni (cf. Supplementary Appendix for details). A heat treatment temperature of 1,423 K (> 0.8 × the melting temperature of all compositions) was selected.

## Results and discussion

### Phase characterisation

**Figure 1:**
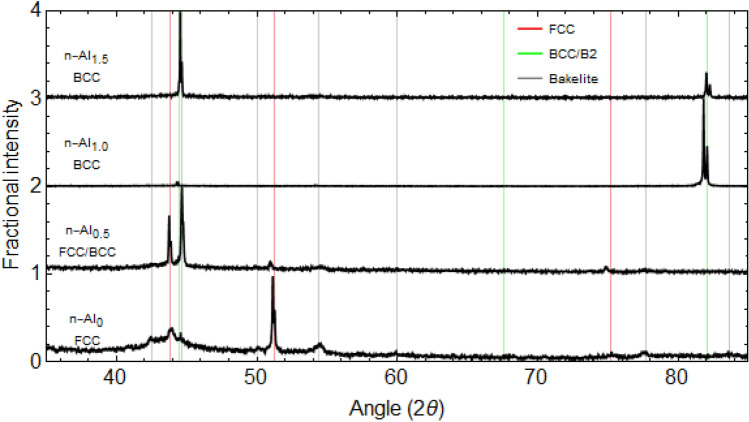
XRD data of the CoFeNi_0.5_Cr_0.5_Al_x_ samples.

The samples were synthesised, heat treated, and prepared for characterisation as described in the Methodology section and initial XRD characterisation was performed. The phases present in the samples were determined from the XRD data (Fig. 1).

The n-Al_0_ sample has a broad peak at 2θ = 43.8°, with two further peaks at 2θ = 51.2° and 77.6° associated with a FCC phase (indicated as red lines). Additional peaks are observed in the sample; as the sample has been etched in HF, it was thought that there was a possibility for the Bakelite to have reacted with the etchant. In order to confirm this, an XRD trace of the n-Al_0_ sample with a Si wafer covering the mounted sample was performed. The FCC peaks were found to disappear, whilst peaks at 42.5°, 44.7°, 50°, 54.4°, 60°, 77.7°, and 86.3° remained—this peaks are indicated by the grey lines (Determination of these are show in Supplementary Fig. A1 in the supporting information). Similar peaks are observed in the n-Al_0.5_, n-Al_1.0_, and n-Al_1.5_ compositions although their low intensities mean that they cannot be easily distinguished in the combined plot above. For the n-Al_0.5_ sample, it is observed that there are two phases present, a FCC phase and a BCC phase (peaks indicated by the green lines). The BCC phase has similar peak positions to the BCC phases observed in the n-Al_1.0_ and n-Al_1.5_ samples and the FCC phase has the same peak positions for the FCC phase found in the n-Al_0_ sample. This means that the Al addition has started to help form the BCC phase, but the concentration is not high enough to achieve a full BCC phase. These results are in agreement with the predictions from modelling^32^ (Fig. 1) and with the work by Wang^12^ where the addition of Al promotes the BCC phase. Using Bragg’s Law the lattice constant of each phase were determined and are given in Fig. 1. The lattice constants are in good agreement across the samples for the FCC phase (n-Al_0_ and n-Al_0.5_) and the BCC phase (n-Al_0.5_, n-Al_1.0_, and n-Al_1.5_). The diffraction patterns suggest that the heat treated samples are highly textured as for each sample certain HKL peaks possess intensity much larger than the other peaks; this may be a residue effect from the arc-melting synthesis process leading to a preferred orientation during grain growth as a result of the cooling kinetics.

### Microstructural characterisation

**Figure 2:**
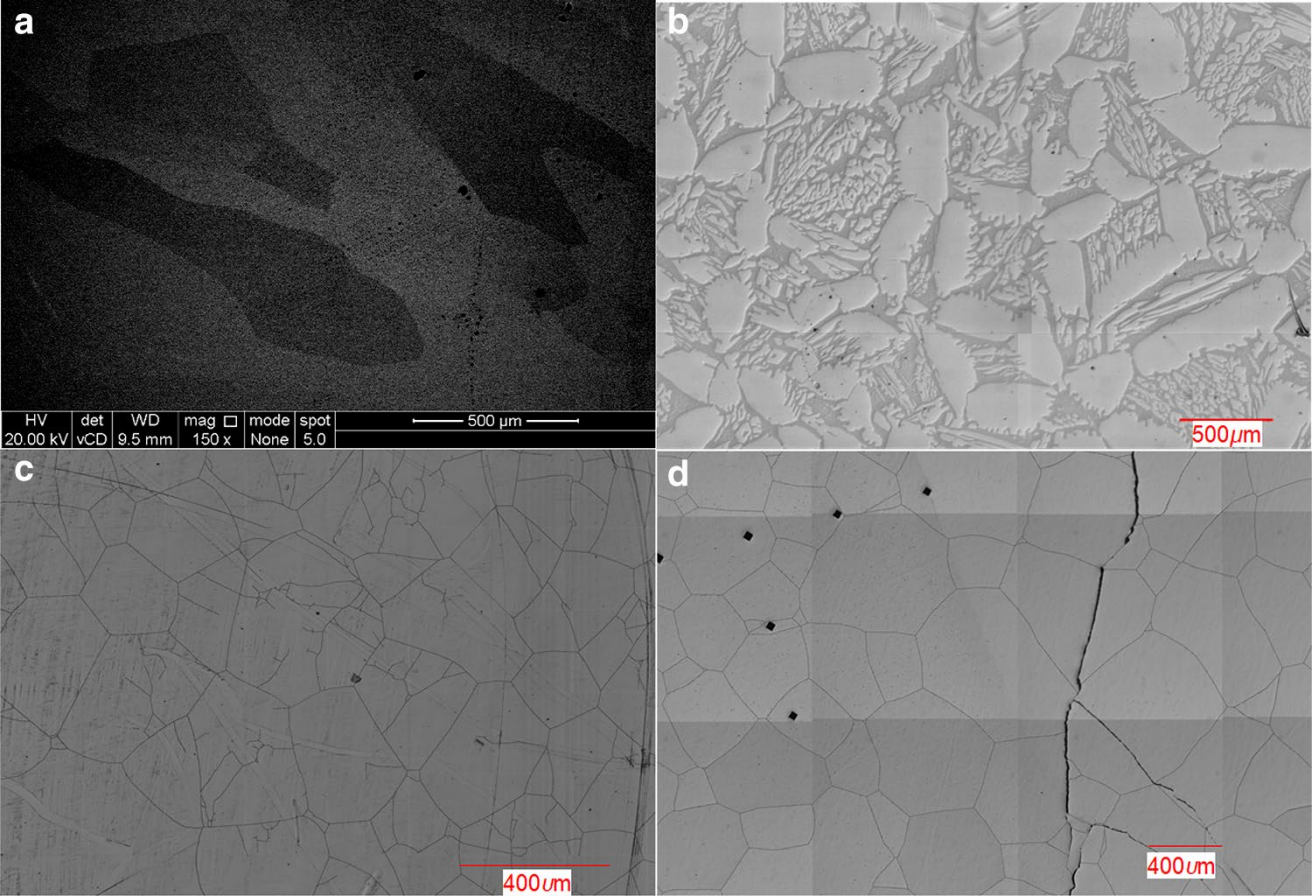
Enhanced micrographs of the heat-treated HF-etched CoFeNi_0.5_Cr_0.5_Al_x_ samples, (**a**) x = 0.0 (SEM), (**b**) x = 0.5 (optical microscopy), (**c**) x = 1.0 (optical microscopy), and (**d**) x = 1.5 (optical microscopy).

Figure 2 indicates the large microstructural features (> 100 μm) observed in the heat-treated samples for n-Al_0_, n-Al_0.5_, n-Al_1.0_, and n-Al_1.5_. The optical micrographs showed no clear distinction between the grains for the n-Al_0_ composition, possibly because the etching process did not strongly distinguish between individual grains as they are of similar phase, requiring enhancement of the grain boundaries. Figure 2 shows the microstructural evolution as a function of Al addition—it is possible to quantify the sizes of the grains (or the larger grains in the case of the n-Al_0.5_ compositions). The n-Al_0_, n-Al_0.5_, n-Al_1.0_, and n-Al_1.5_ micrographs display average grain sizes obtained from image analysis (described in the “Experimental section”) of 692, 206, 196, and 316 µm, respectively, with elongation ratios of 2.31, 1.76, 1.47, and 1.32, respectively. SEM is employed to investigate if the compositional optimisation and heat treatment has resulted in the formation of secondary structures at smaller length scales.

**Figure 3:**
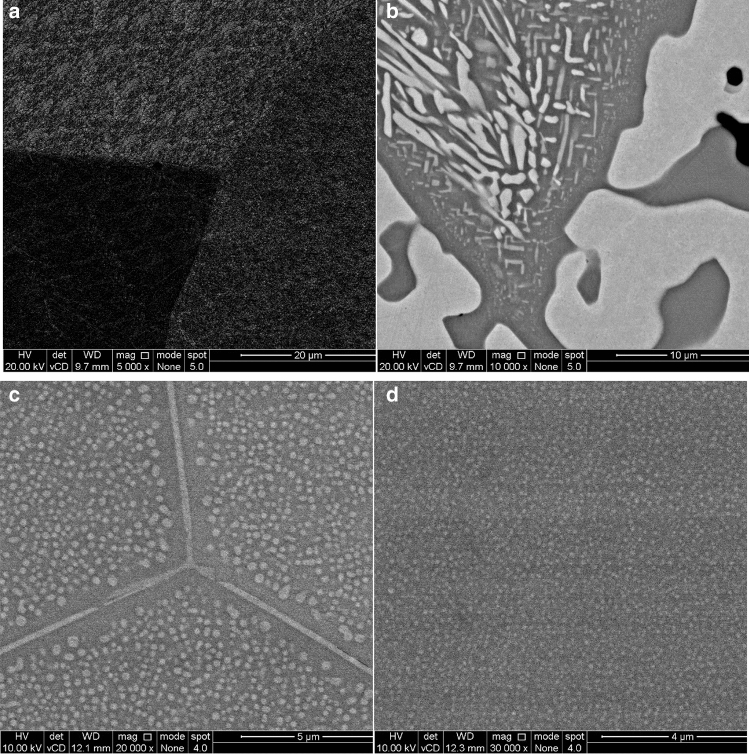
SEM images of the CoFeNi_0.5_Cr_0.5_Al_x_ samples, (**a**) x = 0.0, (**b**) x = 0.5, (**c**) x = 1.0, and (**d**) x = 1.5.

Figure 3 shows a set of micrographs for the n-Al_x_ samples for the length scale in the region of 10 μm. Taken in combination with the previous micrographs of the larger structures (Fig. 2), one can observe that the grain sizes appear to decrease with Al addition up to n-Al_1.0_ with precipitates appearing for Al additions of n-Al_0.5_ onwards. With Al addition, two different phases are observed. In Fig. 3a,b light phase starts to form lath-like grains within a darker phase corresponding to the FCC and BCC phases from the XRD. For the n-Al_0.5_ composition it may be also seen that the lath-like grains within the structure reduce in size depending on the distance away from the larger island-like structures. The literature^12^ suggests that the lighter lath-like structures are likely to be FeCr-rich, with the remaining elements remaining in the darker matrix. The darker Al containing matrix is therefore likely to be the B2 phase as these tend to be stabilised by AlCo and AlNi intermetallic pairs; and it may be inferred from (1) The distribution of varied particles sizes across the sample, and (2) The selected heat treatment temperature argument earlier that the FeCr (likely BCC structure) is precipitated upon heat treatment.

On further Al additions (as in n-Al_1.0_) nanoparticles are observed to be embedded into a matrix (as shown in Fig. 3c) which are homogenously distributed within each grain and circular in shape. In terms of precipitate shapes, for a given precipitate volume a spherical shape has the highest strain energy while a thin plate has the lowest strain energy (this is because almost all metals are mechanically anisotropic)^18,33,34^. As the shape of a precipitate is controlled by the balance between the interfacial energy and the elastic strain energy of the system (i.e. $L = \frac{{\varepsilon^{2} C_{44} r}}{s}$ where *ὲ* is the lattice misfit, C_44_ is the shear modulus, *r* the particle size, and *s* the interfacial energy), this implies that the interfacial energy in the system is high enough to balance the presence of the coherent spherical shapes^35–37^. This is likely due to the n-Al_0_ composition possessing high interfacial energies as large amounts of coherency stresses are required to supress solid solution compositional gradients (from the multiple alloy components)^38,39^.

Noting again that *ΔH* of Al–Co and Al–Ni are much higher than the other binary components, Al addition to the n-Al_0_ system therefore provides a driving force for segregation. Although segregation is generally noted to reduce the interfacial free energy, in this multicomponent system both competing extremes of *ΔH* are presents: (1) Very negative *ΔH* values (< − 15) that drive segregation, and (2) Near-ideal *ΔH* values (0 > *ΔH* > − 5) that lead to compositional gradients. Additionally, magnetic frustration between interfacial Fe–Cr is expected to increase its energy^40^, which may be reflected in the magnetic properties of the nanoprecipitate containing n-Al_1.0_ and n-Al_1.5_ compositions. By corollary, it may be expected for the FeCr precipitates to reduce in size with Al addition as less Fe and Cr are available in the system; this is in line with our experimental data. In order to further test this inference that the phases are split into a FeCr-rich and a AlCoNi phase, we quantify the nanoparticle size and the elemental mixing in the system through EDS analysis.

**Figure 4:**
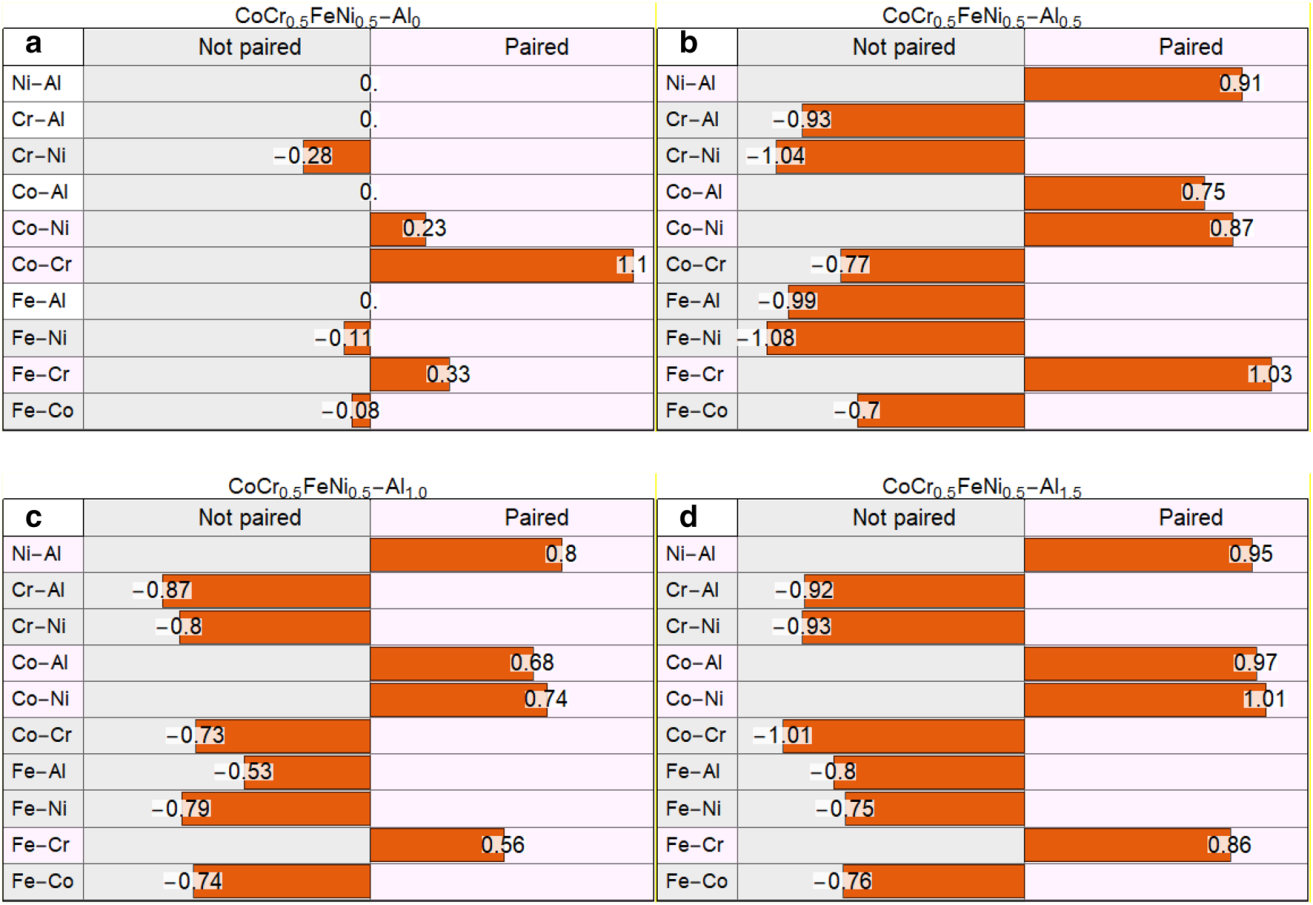
Elemental populations of each EDS line scan was plotted against one another to determine their binary pairing preferences for (**a**) n-Al_0_, (**b**) n-Al_0.5_, (**c**) n-Al_1.0_ and (**d**) n-Al_1.5_. The plots show the gradient for each pair, with positive gradients suggesting pairing and negative gradients suggesting segregation. The plots suggest increased elemental partitioning for n-Al_0.5_.

As the size of the precipitates were much smaller than the spot size of the EDS, an exact elemental distribution could not be obtained. However, analysis was performed utilising a line scan; analysis of elemental pair fluctuations (cf. Fig. 4) reveal that the AlCr, AlFe, CrCo, CrNi, FeCo, and FeNi pairs are not positively correlated with one another. Conversely, AlCo, AlNi, CrFe, and CoNi pairs are strongly linked to one another, suggesting that the compositional mixture is strongly driven by the Al pairs, hence influenced heavily by Al additions. Similar analysis was also performed on the other compositions (n-Al_0.5_, n-Al_1.5_) which showed similar pairing similarities with the n-Al_1.0_ composition. Unsurprisingly, a pair analysis of the enthalpy of mixing values shows that for all the available pairs, the AlCo and AlNi pairs possess the most negative enthalpies of mixing (− 19 and − 22 kJ/mol); other than the binary compounds, AlCoNi stoichiometries have been reported in literature^41^. These results are in excellent agreement with the hypothesis that the AlCoNi and FeCr are segregated.

Image analysis as outlined in the Experimental Section was performed to determine the size distribution of the nanoprecipitates. n-Al_0.5_ has nanoprecipitates which are not uniformly distributed and its analysis is described in the Appendix. The average (mean) size of the precipitates appears to change from 245 nm (length) for the n-Al_0.5_ sample to 180, and 120 nm (diameter) for the n-Al_1.0_, and n-Al_1.5_ alloys. The size of the precipitates thus decreases as a function of Al addition. Figure 1 provides an overview of the samples studied, along with the structural parameters determined from XRD and SEM. The experimental observations of the variation of the particle sizes with the n-Al_0.5_, n-Al_1.0_, and n-Al_1.5_ are in agreement with our inference of FeCr and AlCoNi segregation. We next perform and discuss the magnetic characterisation of the samples in order to validate the hypothesis of increased magnetisation from the alloy design procedure.

### Correlating phase with magnetic behaviour

**Figure 5:**
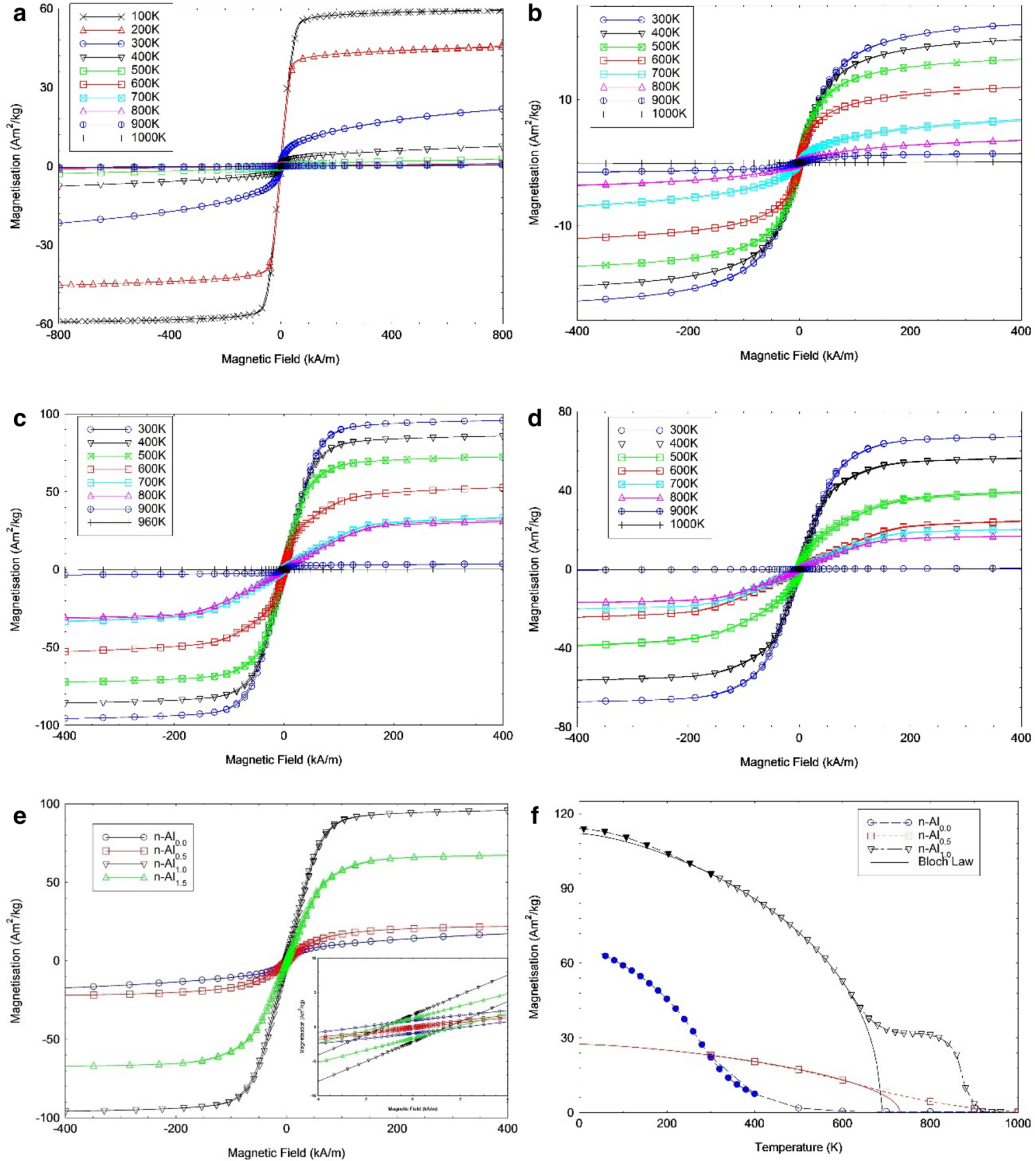
Magnetisation hysteresis loops for the heat treated CoFeNi_0.5_Cr_0.5_Al_x_ samples as a function of temperature, for (**a**) n-Al_0_, (**b**) n-Al_0.5_, (**c**) n-Al_1.0_, (**d**) n-Al_1.5_ measured up to 400 kA/m, (**e**) Comparison of all compositions hysteresis loops at 300 K. Inset: Zoom-in of the hysteresis loops around 0 kA/m to focus on the coercive field, and (**f**) M–T curves at 400 kA/m extrapolated using Bloch’s law to low temperatures from the hysteresis loop data, open shapes represent the high temperature data (> 300 K) and closed shapes represent the low temperature data (< 400 K). The solid lines are the Bloch law fit to the high temperature data.

Figure 5 shows the set of hysteresis loops that have been obtained from the various heat-treated samples utilising the MPMS-3 SQUID. From these loops the total magnetisation at 400 kA/m was determined as a function of temperature. From Fig. 5, it is seen that all the samples have soft magnetic properties, with coercive fields less than 2.5 kA/m at 300 K (Fig. 5e inset). The n-Al_0_ sample has a large paramagnetic component in addition to a magnetic component (Fig. 5a), as the magnetisation does not saturate at high applied magnetic field. From low temperature measurements (100–400 K), the loops become fully saturated at 200 K, with a saturation magnetisation of 58 Am^2^/kg at 400 kA/m measured and 100 K.

**Figure 6:**
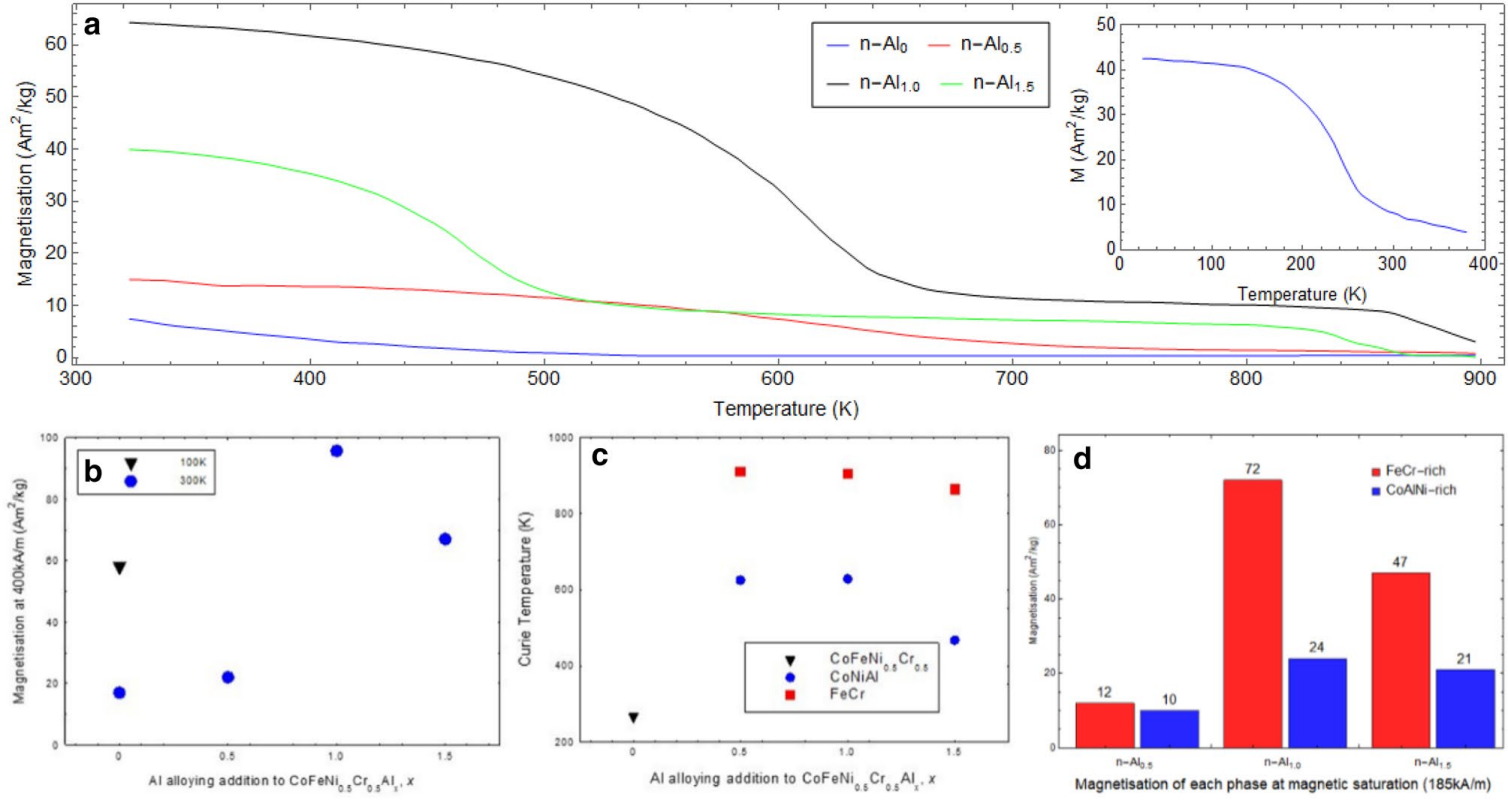
(**a**) Field cooled magnetisation of CoFeNi_0.5_Cr_0.5_Al_x_ samples with an applied field of 48 kA/m for 300 K ≤ T ≤ 1,000 K. Inset: Low Temperature Field cooled magnetisation of CoFeNi_0.5_Cr_0.5_ sample for 50 K ≤ T ≤ 400 K, (**b**) Magnetisation at 100 K (n-Al_0_) and 300 K (n-Al_0,_ n-Al_0.5,_ n-Al_1.0_ and n-Al_1.5_), (**c**) Curie Temperature as a function of Al concentration, and (**d**) Magnetisation of each phase at saturation (185 kA/m).

From these figures, (cf. Figs. 5, 6a), it is observed that with the addition of Al to n-Al_0_, the total magnetisation at 400 kA/m and 300 K increases with Al concentration from 22 to 96 Am^2^/kg, respectively for n-Al_0.5_, and n-Al_1.0_ respectively, and then decreases to 67 Am^2^/kg for n-Al_1.5_. Although precipitates are observed in n-Al_0.5_ onwards (cf. Fig. 3), the median particle size changes from 760, 184, and 100 nm, respectively as more Al is added. This is close to one order of magnitude reduction in median particle size, which suggests that the presence of the FeCr nanoparticles in the n-Al_1.0_ and n-Al_1.5_ samples has an influence on the larger magnetisations observed, as the n-Al_0.5_ samples possess much lower magnetisations at 300 K, 22 Am^2^/kg.

Following our previous inference that AlCoNi and FeCr are separated from one another, we note that the Fe:Cr (2:1) ratios do not change with increasing Al addition. Although Fe:Cr ratios do not change with Al addition, the composition of the matrix does change with Al addition. Referring to Wang et al*.*’s AlCoNi ternary phase diagrams 41 (assuming that the compositional segregation is done well), the phase composition of the matrix shifts from L1_2_ for n-Al_0_ to L1_2_ + B_2_, B_2_, and B_2_ as the Al content increases (L1_2_ and B_2_ are the ordered form of the FCC and BCC phases, respectively). Taking together the analysis of the AlCoNi and FeCr phase diagrams, these observations appear to be in good agreement with our XRD characterisation, and our pre-synthesis predictions of the alloy’s phases (i.e. FCC → FCC + BCC → BCC → BCC) as current semi-empirical models do not allow for the prediction of the ordered FCC/BCC variants (e.g. L_12_, B_2_ etc*.*).

This however, indicates that increasing the Al stoichiometric ratio increases B2 stability, which leads to a corresponding increase in Curie temperature and in saturation magnetisation for fully formed NPs. The increase magnetisation of the BCC/B2 phase (on a phase transition from FCC stability on Al addition) has been previously noted in the literature^42^, which may be driven by increased magnetic ordering, as noted previously^13,43^. However, it is not clear that the changes in magnetic behaviour are solely related to the BCC/B2 structure as extrapolating the MT curves for the n-Al_0.5_ sample using Bloch’s law gives a magnetisation of 27 Am^2^/kg at 5 K (cf. Fig. 5f), which is lower than the measured magnetisation of 66 Am^2^/kg at 5 K for the n-Al_0_ sample. The pronounced drop in M_s_ with Al addition between n-Al_0_ and n-Al_0.5_ can be ascribed to the addition of paramagnetic Al. However, n-Al0 sees a faster drop in Ms in comparison to n-Al0.5 past its Tc (265 K). At temperatures above 295 K, the magnetisation of n-Al0 is lower than n-Al0.5, 19 vs. 23 Am^2^/kg at 300 K. Yet, a significant FM component remains at T > 265 K in n-Al_0_—this may be due to the near-ideal nature of the compositions, which is discussed further below. This variation in magnetic behaviour between n-Al_0_ and n-Al_0.5_ should therefore be attributed to the increase in the alloying addition of non-magnetic Al to the composition. Moreover, M_s_ reduction from n-Al_1.0_ to n-Al_1.5_ is 29 Am^2^/kg, which is an order of magnitude larger than that of the volumetric decrease in FeCr alloying elements. This clearly means that other factors other than the FCC to BCC transition affects magnetisation behaviour here. Therefore, one might expect the change in magnetic properties to be caused by a combination of (1) Elemental segregation from Al addition and the resultant interaction between the AlCoNi matrix and the FeCr precipitates; (2) The decreasing particle size of the FeCr precipitates; and (3) The degree of disorder within the newly formed phases due to its non-ideal nature.

The magnetisation as a function of temperature (M–T) data from 1,000 to 300 K (from 400 to 100 K for n-Al_0_) is shown in Fig. 6a (inset). It is observed that the n-Al_0_ sample has a broad transition which starts at ~ 580 K, reaching saturation at ~ 150 K. The Curie Temperature (T_c_) for CoFeNi_0.5_Cr_0.5_ is 265 K (obtained by taking the M–T derivative). The broad transition may be due to the near-ideal nature of the solid solution, coupled with the heat treatment of the alloys leading to segregation within the system. Due to this, there is the possibility of enrichment within the microstructure—this could explain the broadness in the distribution in the T_C_ from Cr enriched/depleted regions that are nearly identical to one another. We can therefore hypothesise that chromium inhomogeneity through the material leads to a wide distribution of magnetic compositions of different Tc, explaining the long tail in the M–T curve and measurable FM response at temperatures higher than 265 K.

Comparing the calculated Curie Temperature with those found in literature^7,13^, it lies within the CoFeNiCr_x_ system, where for x = 0.9 and 0.7 the T_c_ = 300 and 200 K respectively. Kormann et al.’s^8^ theoretical calculations shows that M_s_ experiences a 68% increase from 0.55 to 0.93 μB (54–91 Am^2^/kg) in CoFeNiCr_x_ on Cr_x_ reduction from x = 1 to x = 0.5 and an increase in T_c_ from 119 to 418 K. However, Kormann’s CoFeNiCr_0.5_-M_s_ of 91 Am^2^/kg is higher than our synthesised n-Al_0_ sample (58 Am^2^/kg, taken from M-H loop at 400 kA/m and 100 K)_._ The difference in these values can be explained by the reduction in Ni content. Overall, experimental and simulation results from literature are in good agreement with the results presented here for the n-Al_0_ sample.

As the magneto-thermal analysis in the region from 50 up to 1,000 K shows a single magnetic transition of the n-Al_0_ sample at 265 K, and considering that microstructural and structural analysis at RT show no evidence of compositional segregation of FeCr, it is assumed that Al addition triggers the phase separation into ferromagnetic-FeCr-rich-nanoparticles and a CoNiAl-rich-matrix. Therefore, the magnetic properties of the n-Al_x_ (x ≥ 0.5) are linked to the nanoparticles nucleation, growth, and assembly. In order to investigate these properties, we take a closer look at the magnetic data to compare these to our initial suppositions on the link between the physical microstructure of the alloys and its functional magnetic properties.

### Magnetic properties of the phases: T_c_

From the magnetisation-temperature data (Fig. 6), it is observed that the n-Al_0.5_, n-Al_1.0_, and n-Al_1.5_ samples all have two temperature transitions, which is in agreement with the hypothesis that the microstructure is divided into AlCoNi and FeCr-rich phases. The first transition occurs between 550 and 650 K, and is a broad transition. The second transition occurs between 860 and 930 K, and is a sharper transition. These transitions may be linked to the phases observed in the different samples. We first discuss the possible attribution of the 900–1,050 K transition to the FeCr-rich phase and later the 500–650 K to AlCoNi-rich phase.

A comparison with the literature suggests that FeCr possesses a T_c_ between 900–1,050 K for Cr < 0.25^44^. FeCr T_c_ dependence on Cr addition is also corroborated by other references, showing a slight increase from Fe T_c_ (1046 K) with less than 5% Cr, following by decreasing T_c_ as Cr percentage increases^32^. Shafranovsky et al*.*^45^ studied different concentrations of FeCr foils and nanoparticles, and found that the T_c_ of Fe_66_Cr_33_ nanoparticles was 843 K. This value agrees with our observed transitions; thus the addition of Al to the system has shifted one of the Curie temperatures to higher values through inducing elemental segregation in the system. The T_c_ of the FeCr phase decreases with increasing Al concentration (Fig. 6c). Generally, Cr reduction (here due to Al addition) in FeCr is associated with increasing T_c_, which is not our observation. Although the nanoparticles can be inferred to be FeCr from our pair analysis, it is important to note that in all compositions the Fe:Cr ratio is constant at 1:0.5—this means that any change in T_c_ should be independent of the compositional change. However the FeCr T_c_ drops from ~ 912 K, ~ 900 K, and ~ 862 K from n-Al_0.5_ to n-Al_1.0_ to n-Al_1.5_. This is a 60 K drop for a 3% reduction in Cr alloy content. Taking a linear fit from Shafranovsky et al*.*’s data^45^ suggests an increase in T_c_ of 18 K for a 3% decrease in Cr content—this is in the other direction that we would expect from FeCr effects. The trend we observe is also consistent with Al addition, meaning that experimental error in terms of synthesis and alloy weight-out can be discounted. The decrease in T_c_, we infer to be related to two possible reasons: (1) The size of the FeCr grains/particles decreases with increasing Al concentration (Fig. 3) and this may cause the decrease in the T_c_. (2) Due to small changes in stoichiometric either in the matrix or the precipitates.

Point 2 is explained by the stoichiometric ratios of Al:Co (0, 0.5, 1, and 1.5) and Al:Ni (0, 1, 2, and 3) for n-Alx (x: 0, 0.5, 1, and 1.5) and the binary enthalpy of mixings Al–Cr (− 10 kJ/mol), Al–Co (− 19 kJ/mol), Al–Ni (− 22 kJ/mol), Cr–Ni (− 7 kJ/mol), Cr–Co (− 4 kJ/mol), and Fe–Cr (− 1 kJ/mol). Given Co Fe Cr Ni Al alloying additions, the presence of Al is the limiting factor as Al–Co and Al–Ni have the most negative enthalpies of mixing. When there is not enough Al to pair with Ni and Co, the Ni–Cr and Co–Cr pairs can form since they are the more negative compared to Fe–Cr. When there is enough Al, the phases are fully segregated. However, with excess Al, Al–Cr pairs can form. A reduction in FeCr T_C_ can therefore be explained by Al interacting with the FeCr-rich phase as excess Al is added to the system. This appears to agree with the experimental results as T_C_ initially rises with Al addition but then decreases.

**Figure 7:**
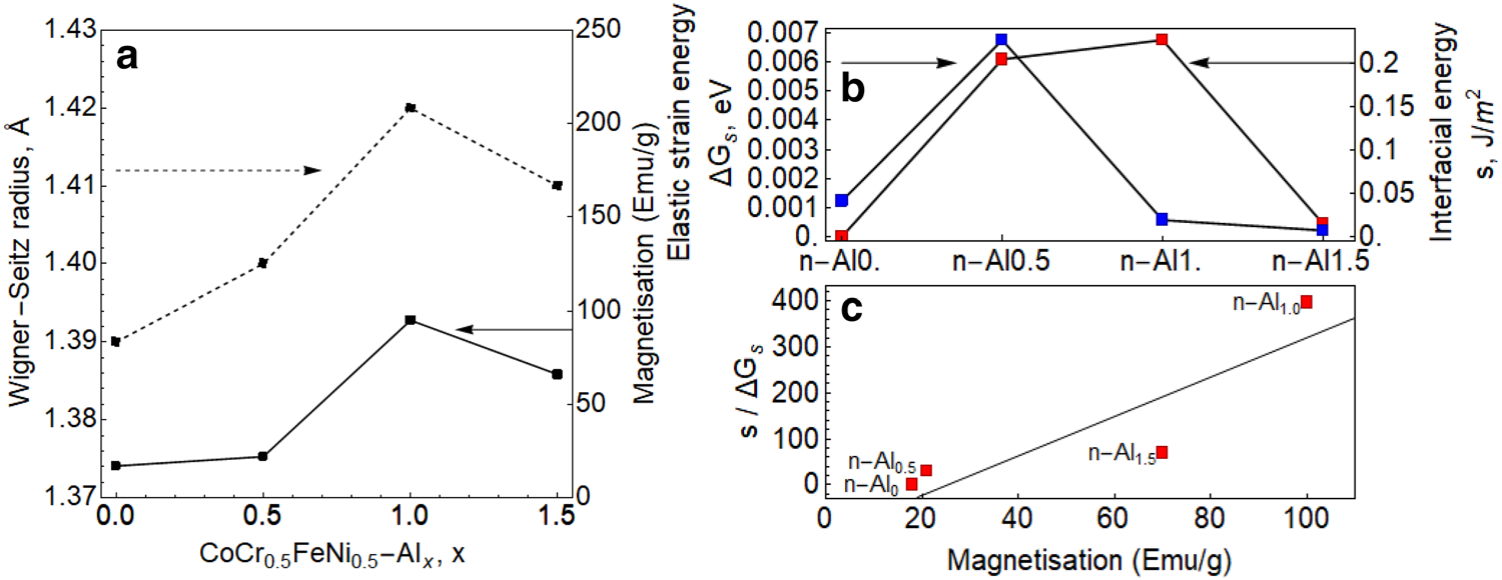
(**a**) Comparison of Wigner–Seitz radius determined from XRD phase fractions and lattice parameters with magnetisation at 400 kA/m values, (**b**) Elastic strain energy, ΔG_s_ and interfacial energy, s plotted as a function of Al addition showing excess s for n-Al_1_, and (**c**) s/ΔG_s_ ratio plotted against magnetisation at 400 kA/m. The line is present as a guide for the eye.

For the 500–650 K transition, the temperature ranges over which it occurs decreases with the increase of Al addition from n-Al_0.5_ to n-Al_1.5_; the n-Al_0.5_ possesses a broad transition of ~ 215 K compared to 80–100 K for the n-Al_1.0_ and n-Al_1.5_ samples. From the literature it is possible to piece together, different T_c_ for different ratios of CoNiAl. From Saito’s paper^46^, they studied Co–Al ribbons with different concentrations of Ni and found that a non-equilibrium BCC phase of (Co,Ni)Al was achieved with a Curie Temperature between 700 K (0% Ni) to 300 K (40% Ni). Other examples of binary alloy T_c_ are Al_0.25_Ni_0.75_ (420 K), Al_56_Co_44_ (180 K), and Al_42_Co_58_ (426 K), all of which tend to be much lower than 900 K. This is backed up by Ikedia’s paper^47^, which on average shows that an increase in the Ni content into CoAl decreases the Curie Temperature, with 20% Ni having a Curie Temperature of 508 K. The concentration of the Ni in this work is between 16.5 and 25% depending on the Al concentration. From Saito’s paper this gives Curie Temperatures between 500 and 700 K, which is observed in Fig. 7. This is in line with our supposition that each transition is representative of a particular region in the microstructure.

Na et al*.*’s paper^7^ studied CoFeNiCr and CoFeNiCrAl, and found that the CoFeNiCr alloy was paramagnetic at room temperature, with a T_c_ = 104 K and a saturation magnetisation of 0.5 Am^2^/kg. For the CoFeNiCrAl alloy, the T_c_ was 277 K and the saturation magnetisation was 25 Am^2^/kg. In our work, we reduced the Ni and Cr percentage in the alloys and this has improved the magnetic properties as all the alloys we measured were magnetic at room temperature with a T_c_ > 400 K. While the saturation magnetisation of the n-Al_0.0_ alloy was a factor of 50 larger than Na’s CoFeNiCr alloy and the n-Al_1.0_ saturation magnetisation was a factor 4 larger than Na’s CoFeNiCrAl alloy.

### Magnetic properties of the phases: M_s_

The magnetisations presented in Fig. 6 were determined using the sample’s total mass (i.e. $M = \frac{moment}{{total\ mass}}$). Based on Fig. 6a the increase in magnetisation for the AlCoNi T_C_ is much larger than for the FeCr T_C_. Our earlier analysis shows that between 700 and 900 K the FeCr-rich phase is the only magnetic component (the AlCoNi-rich phase appears to be paramagnetic at this temperature as *per* the above discussion). From Fig. 6a, the 300 and 700 K magnetisations at 48 kA/m for n-Al_1.0_ are 65 and 13 Am^2^/kg respectively; implying that the AlCoNi-rich and FeCr-rich phases magnetisations are 52 and 13 Am^2^/kg respectively. At first glance this implies that the AlCoNi phase appears to be more magnetic than FeCr. However, the FeCr-rich phase contributes less than half the total mass of the sample at 700 K, but all the measured magnetic moment, as the temperature is higher than the AlCoNi phase T_c_. Thus, the presented magnetisations are misleading in this temperature range. Analysis is non-trivial as both phases contribute to the magnetic behaviour at lower temperatures making it difficult to study each phase in isolation. One possible solution is to take the measured magnetic moment above the AlCoNi-rich T_c_ where only the FeCr-rich phase contributes magnetically, and determine the FeCr phase saturation magnetisation by dividing by the FeCr mass. This value can then be subtracted from the total magnetisation in order to evaluate the contribution from each phase.

We therefore do the following: the magnetic moment (in Am^2^) of the FeCr-rich phase in the n-Al_0.5_, n-Al_1.0_, and n-Al_1.5_ samples at 700 K and 185 kA/m were divided by the mass fraction of the phase using a rules-of-mixtures approach to determine the saturation magnetisation of the FeCr phase. The values were found to be 12, 72, and 47 Am^2^/kg respectively (cf. Fig. 6d). This suggests that the FeCr-rich nanoparticles/phase dominate the total saturation magnetisation of the n-Al_1.0_ and n-Al_1.5_ samples. As discussed, a rough estimate of the saturation magnetisation of the CoNiAl phase can be calculated from the total magnetisation of the sample by subtracting the FeCr saturation magnetisation. This gives 10, 24, and 21 Am^2^/kg respectively for the n-Al_0.5_, n-Al_1.0_, and n-Al_1.5_ samples. The results suggest that the matrix is weakly magnetic compared to the FeCr nanoparticles.

The Ni content influences the magnetisation magnitude as with lower Ni:Co and Ni:Al ratios^48^ the larger the magnetisation observed; this trend corresponds with increasing Al addition. This is also observed in Fig. 6a, where the n-Al_0.5_ has a lower total saturation magnetisation at 300 K compared to n-Al_1.0_, and n-Al_1.5_.

The n-Al_1.0_ saturation magnetisation for the FeCr nanoparticles also correlates well with the values given for the FeCr-rich nanoparticles^45^. (1) The reduction in the saturation magnetisation of the n-Al_1.5_ sample compared to the n-Al_1.0_ ties in with the reduction in the Curie Temperature; and (2) The large change in the FeCr M_s_ values observed from our results in comparison to the bulk values suggests that either the nanoparticles contain impurities (from the AlCoNi-rich phase) or the particle size strongly influences the saturation magnetisation.

### Mechanism for enhanced magnetisation in n-Al_1.0_

Assuming all Co/Cr/Fe/Ni/Al elements remain in solution, a modification of the Slater-Pauling curve with the Mulliken electronegativity predicts a reduction in saturation magnetic moment per atom with > Al despite dilution of the ferromagnetic Fe/Co/Ni components. From our experimental results, the saturation magnetic moment is maximum at n-Al_1.0_; by assuming FeCr and AlCoNi segregation the modified Slate-Pauling curve predicts that the saturation magnetic moment will be reached at a value of n-Al_0.5_. After considering the lack of d-electrons in Al’s valence orbitals^49^ which contributes to the prediction error; this suggests that phase separation enhances sample magnetisation in this system.

In a similar CoCrFeNi system, spin-driven ordering was observed in an ordered FCC phase (L1_2_), reducing the magnetisation due to antiferromagnetic Cr ordering^13^. Spin-driven ordering may also occur in the n-Al_x_ BCC compositions; in the FeCr B2 system (which is the ordered BCC phase), the ferromagnetic/antiferromagnetic transition occurs from 2.88 Å and below (Wigner–Seitz radius, r_ws_: 1.418 Å)^50^. Figure 7a shows a comparison of the averaged Wigner–Seitz radius of the phases with sample magnetisation values, with both trends although being non-linear are in good agreement with one another. The lattice distortion between the AlCoNi-rich matrix and the Fe–Cr precipitates is analogous to an imposed dilatational strain on the system, reducing the atomic density of the FeCr rich precipitates. The term ‘strain energy’ used here is a mechanical energy term with long range order in the alloy system that is separate from its chemical energies^34^. Furthermore, since (1) In antiferromagnetic Cr decreased sensitivity of the spin-density wave to compensate for strain energies occurs with lattice expansion^51^, and (2) In Yang et al.’s work Cr nanoparticles may transition to ferromagnetism when atomic-scale disorder is present^14^, it is of interest to investigate how the strain energy changes with respect to magnetisation.

The elastic strain energy may be evaluated through the following equation^52^:

1 $$\Delta G_{s} = \frac{2}{3}G \Delta^{2} V_{b} E\left( \frac{y}{x} \right),$$

where *E*(*y/x*) is the elastic strain energy of the precipitate that is dependant on its shape, *G* is the shear modulus of the matrix, and *Δ*^2^ is the volume misfit of the precipitate given by *Δ* = (*v*_*b*_* − v*_*a*_)/*v*_*b*_ where *v*_*b*_ is the volume of the unconstrained precipitate, and *v*_*a*_ is the volume of the unconstrained matrix hole. Thus, the magnetisation may be studied as a function of the nanoprecipitates observed in the microstructure. The equilibrium shape of a precipitate is the result of balancing between the elastic strain energy and interfacial energy, which may influence magnetic behaviour^53^. The interfacial energy may be evaluated as a function of the coherent precipitate shape, *L*^54^:

2 $$L = \frac{{\varepsilon^{2} G r}}{s},$$

where *ε* is the lattice misfit between the particle and matrix, *r* is the particle size, and *s* is the interfacial energy. Via our experimental results, the mathematical relationships provide a way to use the equations as numerical laboratory to relate the relative changes in elastic strain energy and interfacial energy with magnetisation behaviour. In this analysis, the shear modulus is approximated by taking the rules-of-mixtures from first-principles calculations of the pure elements^55^, the lattice and volume misfit is obtained from converting the matrix XRD lattice parameters and Fe_2_Cr precipitate volume in Ref.^55^ to Wigner–Seitz radii, *E*(*y*/*x*) and *L* are constants dependant on the particle shape where L = 1 and *E*(*y*/*x*) = 1 for spherical precipitates, L = 10 and *E*(*y*/*x*) = 0.75 for lath-like precipitates, and the particle size is determined from the SEM analysis.

Calculations of *s* and *ΔG*_*s*_ are shown in Fig. 7b showing that the strain energy, *ΔG*_*s*_ is maximum for n-Al_0.5_, whilst the interfacial energy is maximum for n-Al_1.0_. The nucleation of a secondary phase proceeds when the elastic strain energy is sufficient to overcome the chemical potential of the new phase^33^, which may explain the excess elastic strain observed in n-Al_0.5_ as the BCC phase is stabilised then. Changes in interfacial energy is normally expected to correspond to changes in the elastic strain energy as they balance each other out, and the increase in n-Al_1.0_ is unexpected. Plotting *s*/*ΔG*_*s*_ against the magnetisation at 400 kA/m suggests that an increase in magnetisation occurs as long as the change in interfacial energy exceeds the change in elastic strain energy. As the interfacial energy in the system can be affected by magnetic behaviour, it may be that the microstructure may have Cr-depleted/enriched regions for certain compositions as the EDS line scans show possible Al-Cr separation for both n-Al_0.5_ and n-Al_1.5_ (cf. Supplementary Fig. A6) but is much lower for n-Al_1.0_. Further studies on the magnetic structure will be required to understand the complex behaviour exhibited here, such as in-depth numerical analyses on the interfacial and strain energies and better magnetic characterisation.

## Conclusions

The designed reduction in the Ni and Cr content here to form CoFeNi_0.5_Cr_0.5_–Al_x_ alloys has led to a rebalancing of the AlNiCo and FeCr stoichiometries, reducing the FCC stability and therefore enhancing the BCC/B2 stability. Experimental results suggest that the competition between both phases lead to the formation of FeCr-rich and AlNiCo-rich phases. At higher Al additions, an AlCoNi-rich matrix with FeCr spherical nanoprecipitates were observed. The size of the nanoprecipitates appear to scale with Al addition, although grain sizes appear to reach a minimum for n-Al_1.0_ (196 µm) before increasing again with further Al addition. These results are in agreement with the inference of the importance of the AlNiCo–FeCr balancing and suggests that Al addition initially maximises the grain boundary energy before reaching some critical threshold.

From the magnetisation data, two Curie Temperatures were measured, which were associated with the two different phases. It was also found that the addition of Al increased the overall magnetisation of the sample, while maintaining the soft magnetic properties for all temperatures. It was also found that decreasing the concentration of Ni and Cr within the sample increased both the saturation magnetisation and the Curie Temperature. The fact that the FeCr nanoprecitates appear to continue reducing in size suggests that whilst the microstructural behaviour is may be naively described through Al additions, the magnetic behaviour is affected by a combination of both the inter-phase interactions and nanoprecipitate size.

These results demonstrate the ability to design functional new complex concentrated alloys by utilising already-known concepts in the literature.

## Supplementary information

Supplementary Information.
