# Supplementary material for: Magnetic properties of the complex concentrated alloy system CoFeNi0.5Cr0.5Alx
Source: Sci Rep. 2020 Sep 2;10:14506. doi: 10.1038/s41598-020-71463-3 (PMC7468281; doi:10.1038/s41598-020-71463-3)
Supplement: Supplementary file 1 — Supplementary Information. [file 41598_2020_71463_MOESM1_ESM.docx]

**Magnetic Properties of the Complex Concentrated Alloy System** **CoFeNi_0.5_Cr_0.5_Al_x_**

N. A. MORLEY,^a,1^ C. R. B. LIM,^1^ J. XI ,^1^ A. QUINTANA-NEDELCOS,^b,1^ Z. LEONG ^*c,1^

^1^Department of Materials Science and Engineering, University of Sheffield, Sir Robert Hadfield Building, Mapping St., Sheffield, S1 3JD,

[^a^n.a.morley@sheffield.ac.uk](mailto:an.a.morley@sheffield.ac.uk)

[^b^a.quintana-nedelcos@sheffield.ac.uk](mailto:ba.quintana-nedelcos@sheffield.ac.uk)

*[^c^z.leong@sheffield.ac.uk](mailto:cz.leong@sheffield.ac.uk) (corresponding author)

**Appendix**

**A. Alloy prediction – bond length distortion and structural stability**

For CCA compositions consisting mainly of 3d and 4d transition metals, it is possible to differentiate between their relative energies by mapping and analysing the bond length distortion in a BCT structure. The c/a ratios of the BCT structure can be varied so that the crystal structure corresponds to the FCC and BCC structures. Further distortions can be used to indicate increased directionality of the d-orbitals, and in this way representing the more ordered structures. The relative change in energy resulting from bond distortion is approximated from *Rose et al.*^1^:

$E_{Rose}=-E_{Orbital} (1+\beta\alpha^{*})e^{-\beta\alpha^{*}}$ (A1)

where $\beta$ is a fitting constant with $\beta=1.16$ ^1^, $\alpha^{*}=\frac{2(r-r_{m})}{\lambda}$ where $\lambda$ is the Thomas-Fermi screening length $\lambda=\left( \frac{9\pi}{4} \right)^{1/3}\left( \frac{4\pi\varphi_{Slater}(r_{m})}{3} \right)^{1/6}$, $r$ is the radius and $r_{m}$ is the mean radius at which $\frac{d{\varphi(r)}^{2}}{dr}=0$. The final parameters are related to orbital wavefunctions which is approximated as Slater-type orbitals ^2^; $E_{Orbital}$ is the orbital energy of the 4s and 3d orbitals which is the integral of $\varphi_{Slater}(r)$. The radial part of a non-normalised Slater-type orbital is:

$\varphi_{Slater}(4s+3d)=\sum_{4s+3d} r^{n_{Q}-1}e^{-\frac{Z-s}{n_{Q}} r}$ (A2)

where $s$ is the atomic screening constant, $r$ is the radius, $Z$ is the atomic number, and $n_{Q}$ is the primary quantum number. The atomic screening constants are obtained from Clementi ^3^ where the effective atomic number, $l=Z-s$. Taking the Born-Oppenheimer principle the energy required to remove all the atoms surrounding the nucleus to an infinite distance is given by^2,4^:

$E_{T}(N\mathcal{)=H(}N)=\sum_{i=1}^{N=Z} -\left( \frac{Z-s}{n_{Q}} \right)^{2}$ (A3)

The energy of the distorted cell is modified by a shift in interatomic distance and the energy associated with a change in spin satisfying the Pauli exclusion principle. This leads to a corresponding change in the potential energy:

$V\left( r \right)=\frac{N_{d} e^{2}}{r}$ (A4)

Applying the Laplacian gives:

$\nabla^{2}V\left( r \right)=4 \pi e^{2} N_{d} dr$ (A5)

Since for $E_{T}\left( N \right)$ there is no distortion, the energy associated with the distortion from r_m_, the distorted energy $E_{Dist.}$ is then:

$E_{Dis.}\left( \delta r_{Dist.},N_{d} \right)=\sum_{i=1}^{d_{1,2,3\ldots5}} (4 \pi)(N_{d} )(\delta r_{d}(d))(\Delta D)+\sum_{i=1}^{N(\uparrow\downarrow)} \Delta E_{Spin}(N_{d})$ (A6)

where $E_{Dist.}$ is given in terms of the $c/a$ orbital distortion, $\delta r_{Dist.}$ and the number of d electrons, $N_{d}$; the first term is the total energy given by the occupancy of each d-orbital ($d_{1,2,3\ldots5}=d_{xy},d_{xz}, d_{yz},d_{x2+y2} and d_{z2} )$ with each orbital possessing energy $E_{T}\left( r_{m},N \right). \delta r_{d.}$ $\delta r_{d}(d)$ is the normalised distance between the occupied d-orbital from $r_{m}$. The second term arises from the energy required to change the sign of the electron spin to satisfy the Pauli exclusion principle. $\delta r_{Dist.}$ is normalised so that $\frac{\delta r_{Dist.}}{\Delta D}=C$, where $\Delta D$ is the change in distortion, and C is a constant related to the ratio of the distortion of an orbital to $\Delta D$. This method was reported and implemented in a thesis^5^, and the difference the FCC/BCC and FCC/Complex phase stabilities can be obtained by taking the energy difference of each distortion. The results shown in Figure A1 are in good comparison with other studies (experimental & modelling).


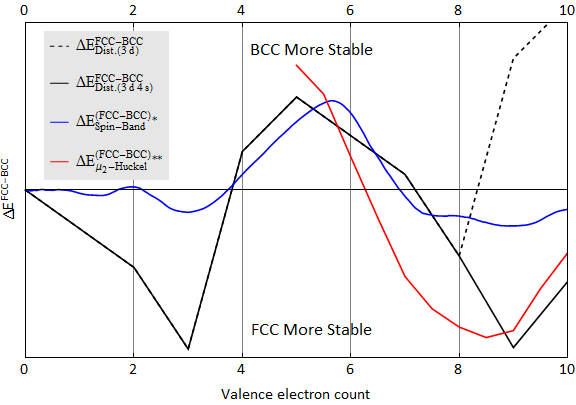

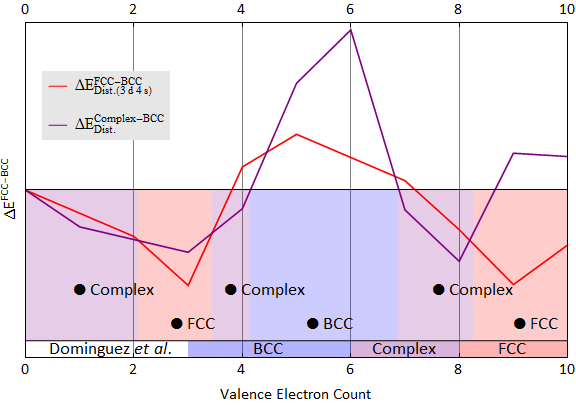


**Figure A1.** A) Plot of the FCC-BCC energy difference calculated using a 3d distorted tetragonal structure, $E_{Dist. (3d)}^{FCC-BCC}$; 4s 3d distorted tetragonal structure, $E_{Dist. (3d 4s)}^{FCC-BCC}$; using the RBA methodology from Chapter 4, $E_{Spin-Band}^{FCC-BCC}$; and adapted from Lee and Hoffman’s tight binding model ^6^, $E_{\mu2-Huckel}^{FCC-BCC}$; B) Plot of $\Delta E_{Dist. (3d 4s)}^{FCC-BCC}$ and $\Delta E_{Dist. (3d 4s)}^{Complex-BCC}$ as a function of the valence electron concentration. Regions of phase stability are highlighted for simple FCC phases (Red), complex phases (Purple), and simple BCC phases (Blue). The phase stabilities as predicted by Dominguez *et al.*^7^ are included at the bottom of the graph, for comparison. Images reproduced from thesis^5^.

The methodology can be further simplified to use inputs from the weighted mean Mulliken electronegativity (X_Mulliken_) and the d-electron count (*n*) and was employed in this work^5^.

$E^{FCC-X}=C+X_{Mulliken}e^{7-n}$ (A7)

where C is a fitting constant with value -11.


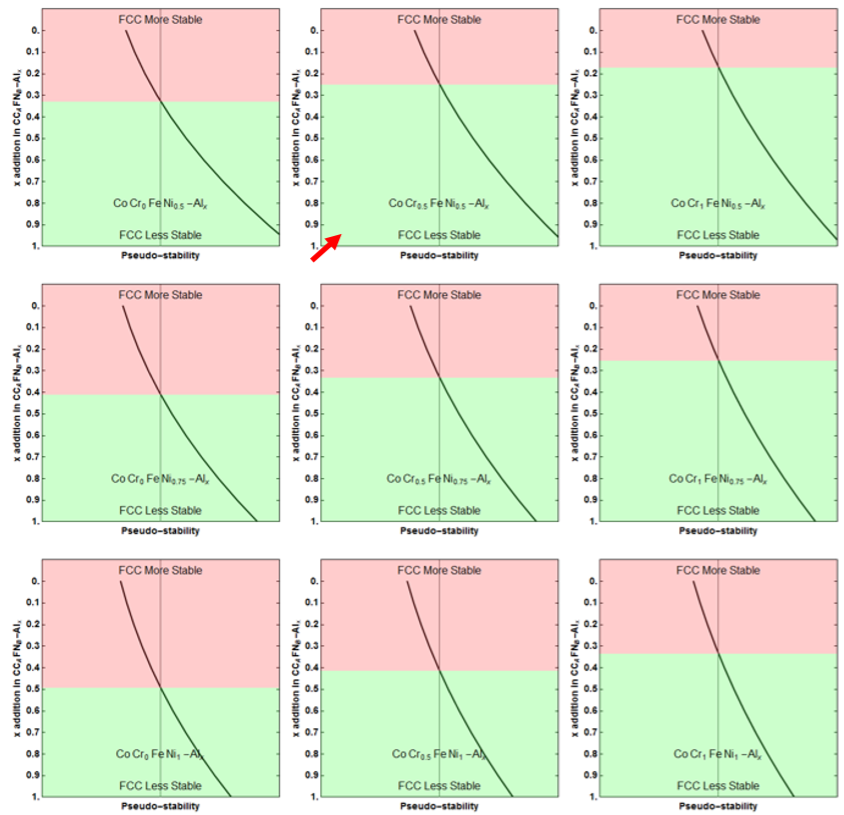


**Figure A2.** Predictions for different compositions with stoichiometry CoCr_a_FeNi_b_-Al_x_

A number of possible compositions were modelled by varying the Cr, Ni, and Al additions in a Cr+Fe+Ni+Co+Al system from 0.5 to 1 in steps of 0.25, with these results shown in Figure A2. The FCC to secondary phase transition points were noted and minimised with respect to the Cr compositional addition. It was found that a good balance between the transition point and the Cr/Ni ratios was obtained from a CoCr_0.5_FeNi_0.5_-Al_x_ (henceforth denoted n-Al_x_) composition. Our predicted results suggest that the FCC structure is destabilised for Al additions above n-Al_0.25_.


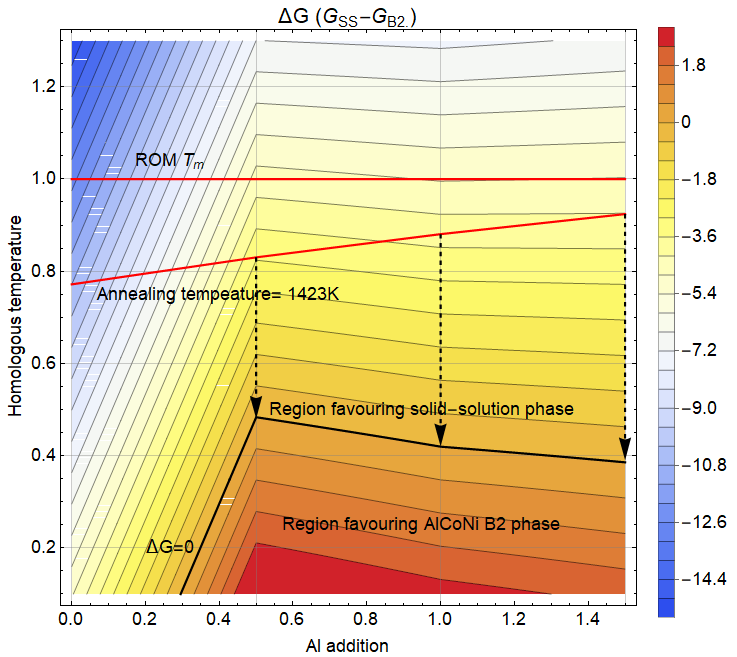

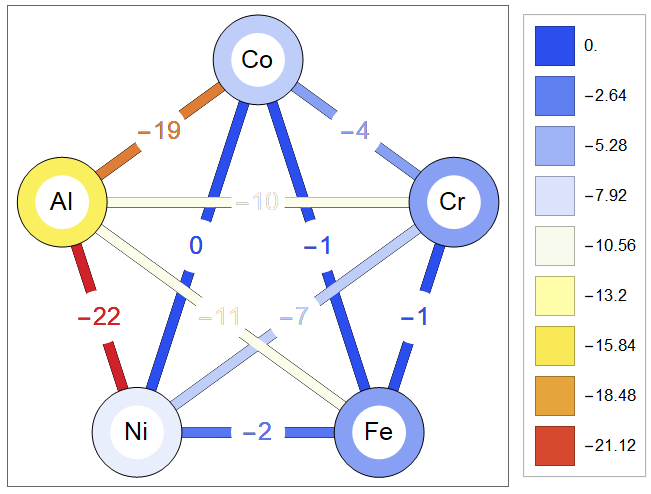


b)

a)

***Figure A3.*** *(a)* ΔG values to distinguish stability regions of the solid solution and the expected B2 phase*;* (b) Binary *ΔH* values of the elemental additions to any Cr+Fe+Ni+Co+Al system

The predictive results used have been confirmed following more commonly used biplot phase prediction schemes used in the literature;^7–9^ here enthalpy of mixing values (binary values are shown in Figure A3) and mean Mulliken electronegativity (*ΔH-X_Mulliken_*) biplots are used to determine phase stabilities. Figure 1 lists equivalent values for each composition that are used for MCA stability predictions. The results show that the BCC structure is stabilised from n-Al_0.5_ onwards, lower than that exhibited by the CoFeCrNi-Al_x_ compositions from x: 1.0 onwards. These results are in good agreement with the predicted results used for the tetragonal distortion scheme.

The results of these predictions appear to vindicate our choice to modify the CoFeCrNi-Al_x_ compositions with reduction in the Cr and Ni additions to the system. Values of x: 0, 0.5, 1.0, and 1.5 are commonly used when investigating new compositions. As the transition point was predicted at n-Al_0.25_, a solid solution/mixed solid solution is expected for n-Al_0_ and n-Al_0.5_, while complex phase precipitates are expected for values of n-Al_1.0_ and n-Al_1.5_. This range of additions allows analysis of the impact of these phase stabilities on the ageing mechanisms of the alloys *via* heat treatment along with their magnetic properties.

**B. Heat treatment temperatures**

Al addition to CoCrFeNi compositions was previously noted to form nanoparticles within the bulk alloy, which lead to the enhancement of ferromagnetic properties.^10^ Heat treatment can help to define the phases (solid-solutions vs. more ordered structures) within the sample compared to the as-cast samples,^10^ so enhancing ferromagnetic properties.

In order to determine suitable heat treatment temperatures and to try and understand the phase stabilities of the fabricated alloys as a function of temperature, we considered the free energy of the solid solution *vs.* that of the B2 structure. The free energy is obtained from these following assumptions:

1. The solid solution phase consists of all alloying compounds in concentrations is equal to the bulk value
2. The complex phase is assumed to be Al-Ni or Al-Co containing B2 structures as the highest binary enthalpy of mixing values arise from these two pairs (*cf.* Figure A3).

Utilising these, the free energy of mixing may be obtained utilising Miedema enthalpy of mixing values obtained from^11^ approximated to a multiple component structure utilising the mean field theory (ΔH_Mix_) and the entropy of mixing (ΔS_Mix_) values from both assumptions taken above. The energy difference between the solid solution (SS) and the complex phase (B2) may then be plotted out as:

$\Delta G=(\Delta H_{Mix}\left( SS \right)-\Delta H_{Mix}(B2))-T(\Delta S_{Mix}\left( SS \right)-\Delta S_{Mix}(B2))$ (A8)

where the temperature is normalised for both structures as the homologous temperature (T/T_M_), where T_M_ is the melting temperature that is determined from the rules of mixtures (ROM).

The results of these calculations are shown in Figure A3 as a contour plot of homologous temperature against Al addition (0 < n < 1.5) in increments of 0.1. They suggest that the compositions progress from FCC to FCC/BCC to BCC/B2 as Al is added as the AlCoNi B2 phase is present with increasing Al additions. The temperatures which correspond to *ΔG_Mix_*=0 are indicated on the plot as a solid black line, with the ROM melting temperature indicated in red. The analysis of the difference between the free energy of the CoFeCrNi-Al_x_ solid-solution and AlNi/AlCo B2 phases as a function of the homologous temperature reveals that the B2 structure is expected to be present for Al additions >0.5, annealing temperatures above ΔG_Mix_=0 would be expected stabilise the solid solution phase.

Decreased ordering in the system is expected to lead to increased magnetic properties and for this reason an annealing temperature of 1423 K was chosen as this is the maximum temperature the furnace being used can reach, and it lies reasonably below the ROM T_m_ of the Al.

**C. Determining peaks not related to the sample**


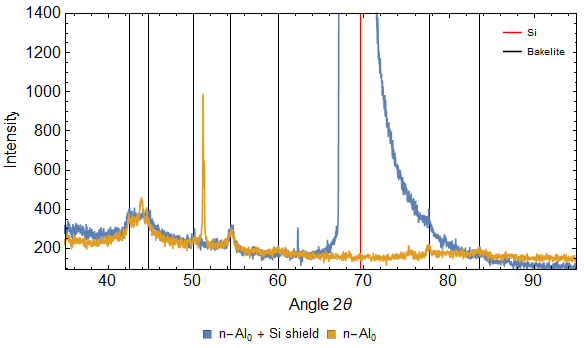


**Figure A4**. Comparison of XRD traces between the mounted sample and the same sample that is shielded with a Si wafer

Determining Bakelite peak formation on HF etching by comparing XRD traces of the mounted sample (42.5, 44.7, 50, 54.4, 60, 77.7, and 53.63^o^ in orange) and the mounted sample with a Si wafer obscuring it (in blue). Si peaks are identified (69.6^o^ in red) and peaks that coexist along both XRD traces are identified as signals arising from the Bakelite mounting. The additional signals suggest that crystallinity has developed through possible oxidation of the Bakelite during the etching process. The remaining unindexed peaks (43.85, 51.22, and 75.24^o^) in the n-Al_0_ sample correspond to the FCC structure. The identified Bakelite peaks are also observed for the n-Al_0.5_, n-Al_1.0_, and n-Al_1.5_ peaks.

**D. Analysis of elemental pairs in the n-Al­_x_ systems**


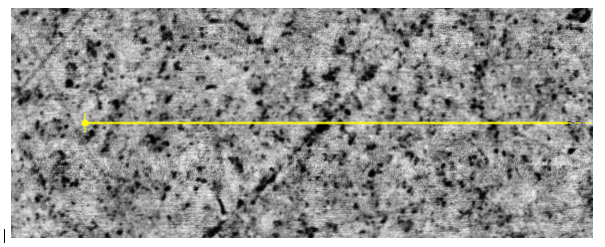

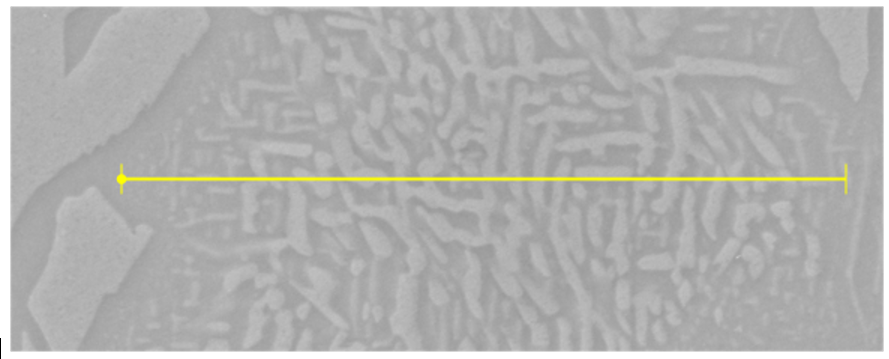

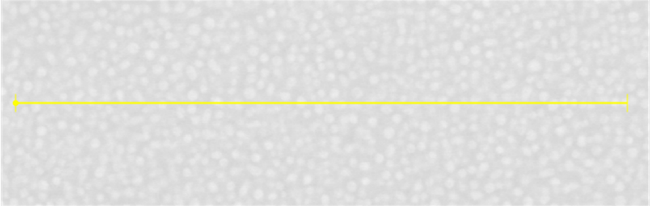

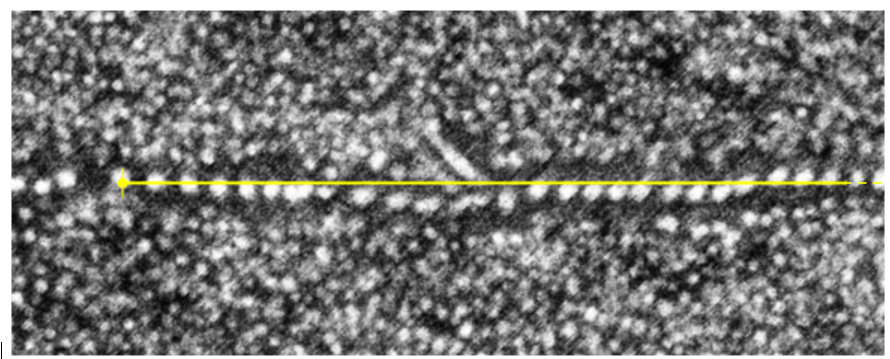


d)

c)

b)

a)

**Figure A5.** Line scans of the different heat-treated as-cast compositions. From top-left a) n-Al_0_, b) n-Al_0.5_, c) n-Al_.0_ and d) n-Al_1.5._

The Figure A5 indicates SEM micrographs of the areas from which EDS line scans were obtained.

The elemental fluctuations of Co, Fe, Cr, Ni, and Al were obtained as a function of distance in the line scan (*cf.* Figure A5), from which it was possible to correlate the fluctuations of each element with each other. In all cases, the line scans were for a length of at least 7 μm. EDS Spatial resolution is approximately 0.04 μm^2^ for a 5kV beam and 2μm^2^ for a 20 kV beam so at least 7 μm is required for subsequent analysis.


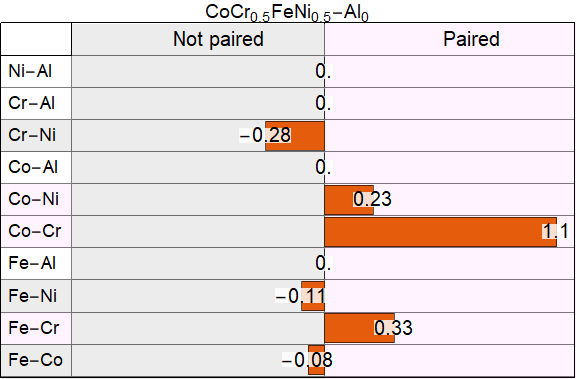

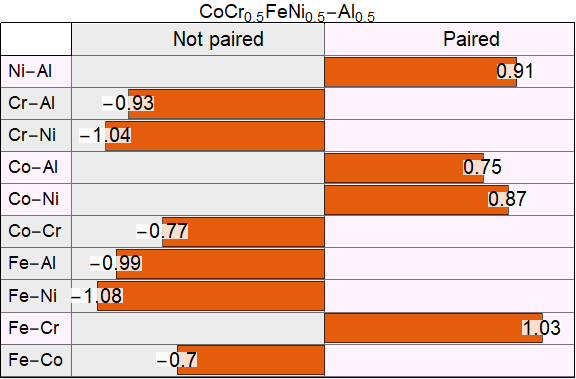


a)

b)


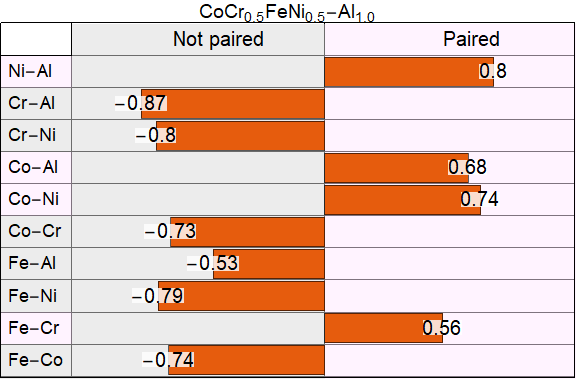

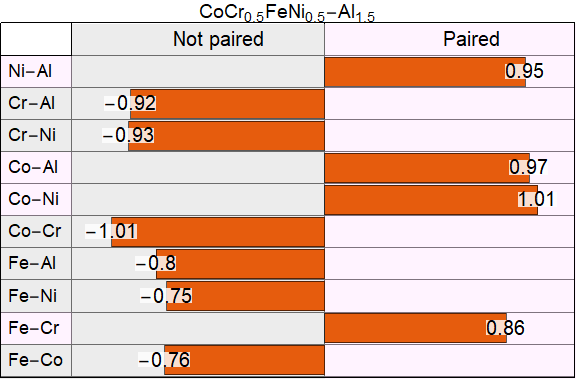


d)

c)

**Figure A6.** Elemental populations of each EDS line scan was plotted against one another to determine their binary pairing preferences for a) n-Al_0_, b) n-Al_0.5_, c) n-Al_.0_ and d) n-Al_1.5_.

An interpolation based schema was developed and used to correlate the pairing of each element with each other from EDS line scans (*cf.* Figure A3 in the supporting information) – an analysis of the gradient of the resulting curve indicates whether each element is paired with another. Figure A6 indicates the pairing of all possible elemental combinations.

The gradient of each biplot was determined and positive gradients implied elemental pairing and *vice-versa*. Very small gradient fluctuations were noted for the n-Al_0_ compositions suggesting that it remained in a semi-ordered solid solution. The reverse is true for the other compositions with Fe-Cr, Co-Ni, Co-Al, and Ni-Al pairs showing positive values. These values mirror the negative enthalpy of mixing values shown by Al-Ni (-22 kJ/mol) and Al-Co (-19 kJ/mol) that appears to drive ordering in the system). When the BCC structures is initially stabilised for n-Al_1.0_ the composition fluctuation appears to be suppressed (comparing this with n-Al_0.5_ and n-Al_1.5_).

**E. Analysis of microstructure size distribution in the n-Al_0.5_ sample**

**
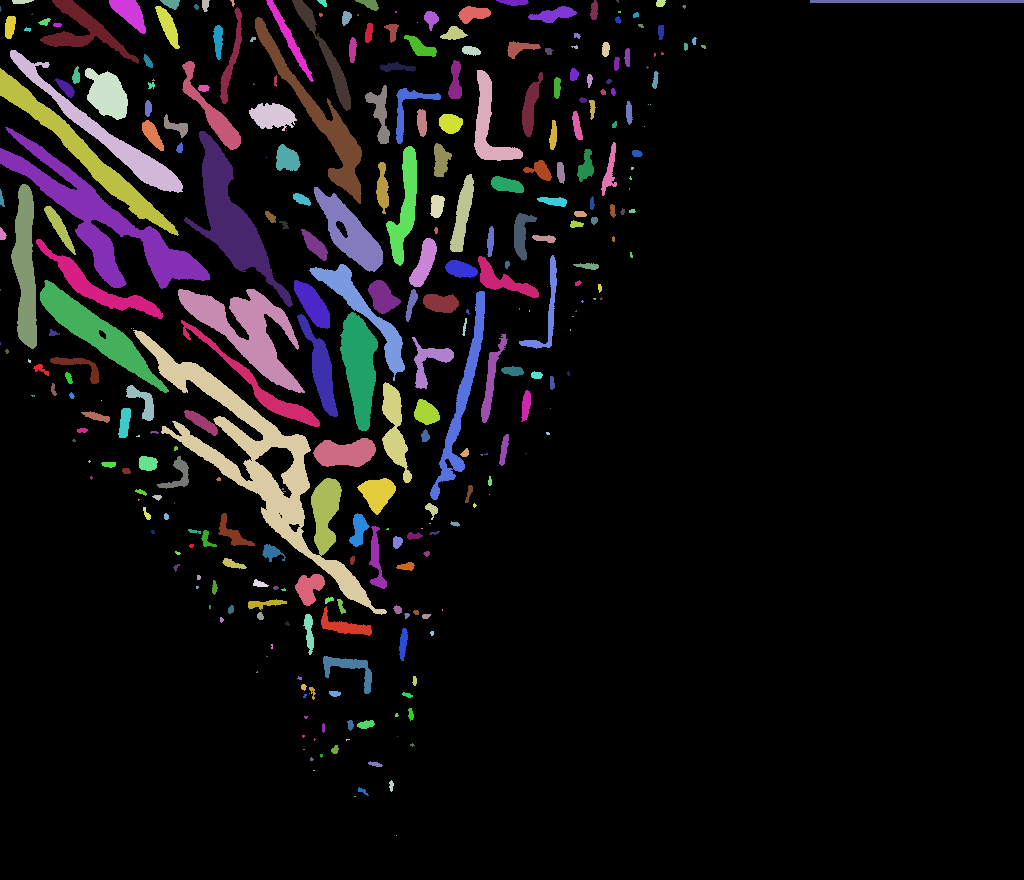

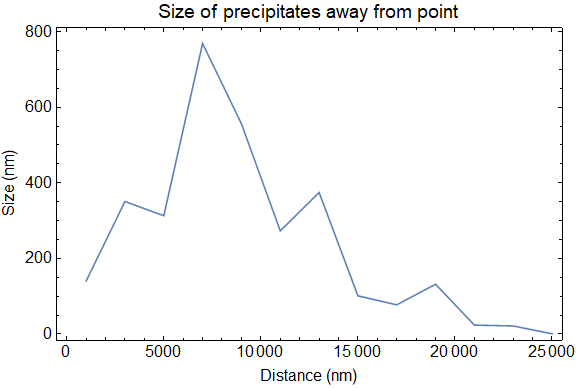
**

a)

b)

**Figure A7.** The morphological components of the n-Al_0.5_ micrograph shown in Figure 3, together with the resulting analysis indicating the size of precipitates from the red point indicated on the micrograph.

Image analysis was performed for the smaller lath-like grain distribution in the n-Al_0_ micrograph. The result of this procedure on the n-Al_0.5_ micrograph is colourised to indicate each morphological component detected. A coordinate list for manipulation and analysis can thus be obtained. The analysis indicates smaller precipitate sizes as the precipitates close in on the larger grains, suggesting that the interfacial energy is much higher for the larger grains. From the information collected from this analysis, the particle size distribution may be analysed. The particle size distribution of the n-Al_0.5_ composition is compared to the precipitate distribution in samples n-Al_1.0_ and n-Al_1.5_ – the results are shown in Figure A8. The precipitates in n-Al_0.5_ have 701 and 245 μm median and mean sizes respectively. This is because there are many more small precipitates closer to the larger grains. In contrast, the median and mean radius for n-Al_1.0_ and n-Al_1.5_ are quite similar, at 91 and 92 nm respectively for n-Al_1.0_ and decreasing to 53 and 66 nm respectively for n-Al_1.5_.

**
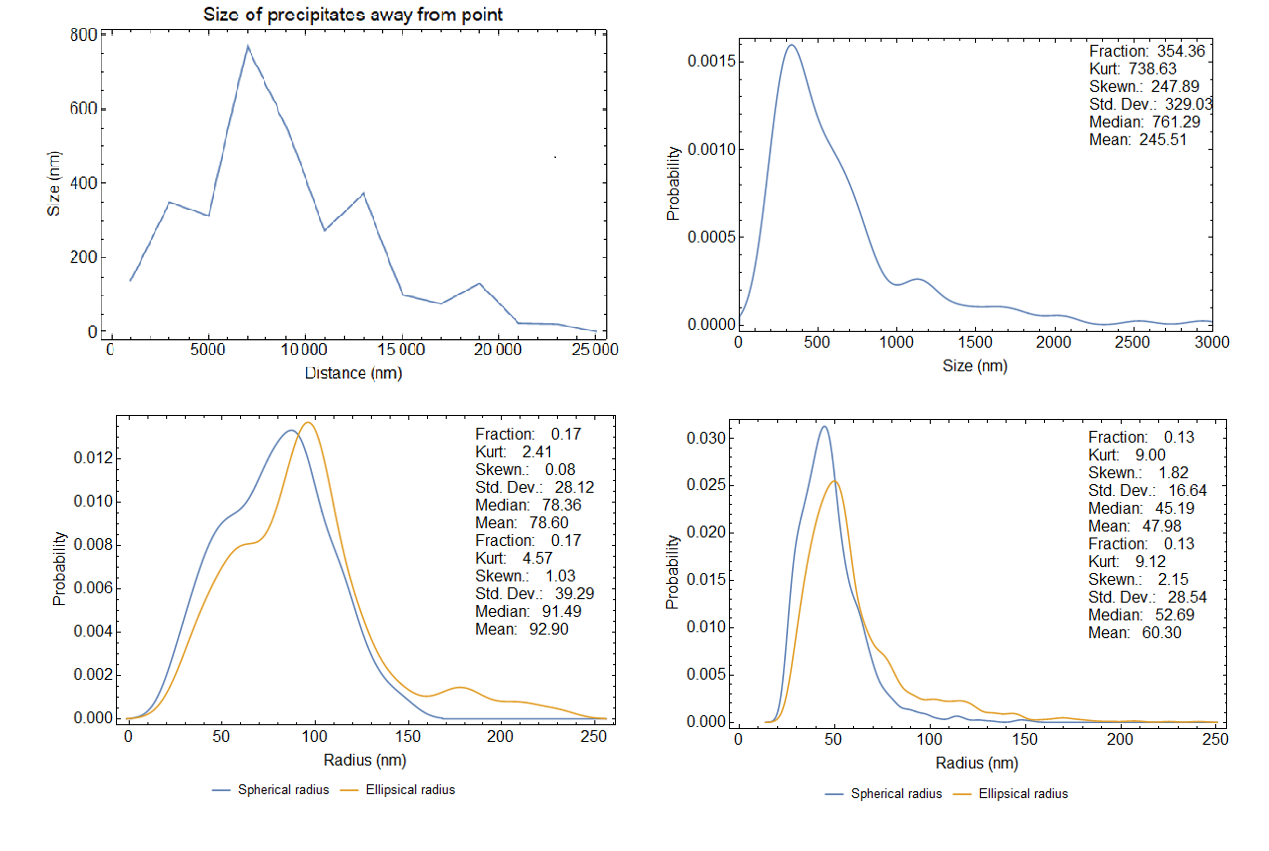
**

c)

a)

b)

d)

**Figure A8.** a) Precipitate size distribution in n-Al_0.5_ determined from the top edge of the micrograph shown in Figure 3b, and the size distribution of the precipitates for the precipitates in b) n-Al_0.5_, c) n-Al_1.0_, and d) n-Al_1.5_

**References**

1. Rose, J. H., Ferrante, J. & Smith, J. R. Universal Binding Energy Curves for Metals and Bimetallic Interfaces. *Phys. Rev. Lett.* **47**, 675–678 (1981).

2. Slater, J. C. Atomic Shielding Constants. *Phys. Rev.* **36**, 57–64 (1930).

3. Clementi, E. & Raimondi, D. L. Atomic Screening Constants from SCF Functions. *J. Chem. Phys.* **38**, 2686 (1963).

4. Zener, C. Analytic Atomic Wave Functions. *Phys. Rev.* **36**, 51–56 (1930).

5. A new semi-empirical method based on a distorted tetragonal scheme for the structure prediction and alloy design of multiple-component alloys. (University of Sheffield, 2017).

6. Lee, S. & Hoffmann, R. Bcc and Fcc Transition Metals and Alloys: A Central Role for the Jahn−Teller Effect in Explaining Their Ideal and Distorted Structures. *J. Am. Chem. Soc.* **124**, 4811–4823 (2002).

7. Dominguez, L. A., Goodall, R. & Todd, I. Prediction and validation of quaternary high entropy alloys using statistical approaches. *Mater. Sci. Technol.* **31**, 1201–1206 (2015).

8. Guo, S., C, N., J, L. & T, L. C. Effect of valence electron concentration on stability of fcc or bcc phase in high entropy alloys. *J. Appl. Phys.* **109**, 10 (2011).

9. Leong, Z., Huang, Y., Goodall, R. & Todd, I. Electronegativity and enthalpy of mixing biplots for High Entropy Alloy solid solution prediction. *Mater. Chem. Phys.* (2017) doi:10.1016/j.matchemphys.2017.09.001.

10. Wang, W.-R., Wang, W.-L. & Yeh, J.-W. Phases, microstructure and mechanical properties of AlxCoCrFeNi high-entropy alloys at elevated temperatures. *J. Alloys Compd.* **589**, 143–152 (2014).

11. Takeuchi, A. & Inoue, A. Mixing enthalpy of liquid phase calculated by miedema’s scheme and approximated with sub-regular solution model for assessing forming ability of amorphous and glassy alloys. *Intermetallics* **18**, 1779–1789 (2010).
